# Strain and its correlates among carers of people with dementia in low-income and middle-income countries. A 10/66 Dementia Research Group population-based survey

**DOI:** 10.1002/gps.2727

**Published:** 2012-03-29

**Authors:** Martin Prince, Henry Brodaty, Richard Uwakwe, Daisy Acosta, Cleusa P Ferri, Mariella Guerra, Yueqin Huang, KS Jacob, Juan J Llibre Rodriguez, Aquiles Salas, Ana Luisa Sosa, Joseph D Williams, AT Jotheeswaran, Zhaorui Liu

**Affiliations:** 1 King's College London, (Institute of Psychiatry, Centre for Global Mental Health, Health Service and Population Research Department)London, UK; 2 Dementia Collaborative Research Centre, School of Psychiatry, Faculty of Medicine, The University of New South WalesSydney, NSW, Australia; 3 Nnamdi Azikiwe University Teaching HospitalNnewi, Anambra State, Nigeria; 4 Universidad Nacional Pedro Henriquez Ureña (UNPHU), Internal Medicine DepartmentGeriatric Section, Santo Domingo, Dominican Republic; 5 Universidad Peruana Cayetano Heredia and Instituto de la Memoria y Desordenes RelacionadosLima, Perú; 6 Peking University Institute of Mental Health, Key Laboratory of Mental Health, Ministry of Health (Peking University)Beijing, China; 7 Christian Medical CollegeVellore, India; 8 Facultad de Medicina Finley-Albarran, Medical University of HavanaHavana, Cuba; 9 Medicine Department, Caracas University Hospital, Faculty of Medicine, Universidad Central de VenezuelaCaracas, Venezuela; 10 The Cognition and Behavior Unit, National Institute of Neurology and Neurosurgery of Mexico, Autonomous National University of MexicoDelegacion Tlalpan, Mexico City, Mexico; 11 Voluntary Health ServicesChennai, India; 12 Public Health Foundation for IndiaNew Delhi, India

**Keywords:** dementia, developing countries, caregivers, stress, psychological, aged, epidemiologic studies

## Abstract

**Objectives:**

In a multi-site population-based study in several middle-income countries, we aimed to investigate relative contributions of care arrangements and characteristics of carers and care recipients to strain among carers of people with dementia. Based on previous research, hypotheses focused on carer sex, care inputs, behavioural and psychological symptoms (BPSD) and socioeconomic status, together with potential buffering effects of informal support and employing paid carers.

**Methods:**

In population-based catchment area surveys in 11 sites in Latin America, India and China, we analysed data collected from people with dementia and care needs, and their carers. Carer strain was assessed with the Zarit Burden Interview.

**Results:**

With 673 care recipient/carer dyads interviewed (99% of those eligible), mean Zarit Burden Interview scores ranged between 17.1 and 27.9 by site. Women carers reported more strain than men. The most substantial correlates of carer strain were primary stressors BPSD, dementia severity, needs for care and time spent caring. Socioeconomic status was not associated with carer strain. Those cutting back on work experienced higher strain. There was tentative evidence for a protective effect of having additional informal or paid support.

**Conclusions:**

Our findings underline the global impact of caring for a person with dementia and support the need for scaling up carer support, education and training. That giving up work to care was prevalent and associated with substantial increased strain emphasizes the economic impact of caring on the household. Carer benefits, disability benefits for people with dementia and respite care should all be considered. Copyright © 2012 John Wiley & Sons, Ltd.

## Background

Alzheimer's Disease International estimates 35.6 million people worldwide living with dementia, of whom 58% hail from low-income and middle-income countries (LMIC). Numbers are forecast to increase to 115.4 million by 2050, much of that increase occurring in middle-income countries (ADI, 2009). In LMIC, the reliability and universality of the family care system is often overestimated with traditional family structures under threat from social and economic changes accompanying economic development and globalization (Tout, 1989; Prince *et al.*, 2008).

Schulz and Martire (2004b) defined caregiving as

‘…the provision of extraordinary care, exceeding the bounds of what is normative or usual in family relationships. Caregiving typically involves a significant expenditure of time, energy, and money over potentially long periods of time; it involves tasks that may be unpleasant and uncomfortable and are psychologically stressful and physically exhausting.’

The onset of caring is hard to define; it emerges naturally from support customarily given and received before the onset of dementia and may precede or post-date a formal diagnosis (Gaugler *et al.*, 2003). Needs for care escalate over time, from support for instrumental activities of daily living (IADL—household, financial and social activities), to personal care, to what may be almost constant supervision and surveillance (Schulz and Martire, 2004a). According to a recent review, carers of people with dementia spend an average of 1.6 h daily assisting with personal care, a total of 3.7 h having included IADL or 7.4 h per day when supervision was included (Wimo *et al.*, 2007). In high-income countries (HIC), important transitions include involvement of professional carers, institutionalization and bereavement. The negative consequences of caregiving have been widely studied (Sorensen *et al.*, 2006). Nevertheless, many informal carers take pride in their role and perceive many positives (Cohen *et al.*, 2002).

There are few studies of carers of people with dementia from LMIC. The 10/66 Dementia Research Group's multicentre pilot study included 706 carers in Latin America, India and China (10/66 Dementia Research Group, 2004), with a methodology that was similar to the EUROCARE study of 280 spouse carers from 14 European countries (Schneider *et al.*, 1999). Both studies recruited convenience samples, so findings may not have been representative. In both studies, most carers were women. In Europe, most couples lived on their own. In the 10/66 studies, people with dementia typically lived in large households with extended families, one-quarter to one-half of households including children under 16 years of age (10/66 Dementia Research Group, 2004). Whereas larger households attenuated slightly the strain for the main carer, traditional extended family care networks provided little protection; levels of carer strain were, generally, still as high as in the EUROCARE project.

In HIC female carers (Yee and Schulz, 2000; Gallicchio *et al.*, 2002), spouses (Brodaty and Hadzi- Pavlovic, 1990), carers that live with the care recipient (Brodaty and Hadzi-Pavlovic, 1990), and those with low incomes or financial strain (Schneider *et al.*, 1999; Covinsky *et al.*, 2003; Andren and Elmstahl, 2007) are prone to high levels of strain or distress. Behavioural and psychological symptoms of dementia (BPSD—particularly apathy, irritability, anxiety, depression and psychosis) are strongly associated with carer strain (Pinquart and Sorensen, 2003). Cognitive impairment is less strongly implicated (Pinquart and Sorensen, 2003). Others' availability to assist in providing care may be more important than non-specific indices of social support (Chang *et al.*, 2001). Coping strategies may mediate associations between carer subjective strain and psychological morbidity (Pruchno and Resch, 1989; Cooper *et al.*, 2008). Finally, poorer relationship quality and lack of past intimacy have also been reported to be associated with increased strain.

In the context of the 10/66 Dementia Research Group cross-sectional population-based surveys of dementia and ageing in Latin America, China and India (Prince *et al.*, 2007), we aimed to investigate in each site, among those survey participants diagnosed with dementia, the relative contributions of care arrangements and characteristics of carers and care recipients to carer strain and to estimate the effect of site on carer strain, controlling for these factors. Specifically we aimed to test the following hypotheses

1. Mean carer strain will be as high as that observed in previous HIC studies.
2. Female and spouse carers will report more carer strain.
3. Carer strain will be associated with time spent caring and with BPSD more than with cognitive impairment.
4. Socioeconomic status (assets and higher education levels of carers and care recipients) will be independently inversely associated with carer strain, whereas giving up work to care will be associated with higher strain.
5. Those who employ paid carers will experience less strain, as will those reporting availability of alternative informal carers.
6. Variation in mean carer strain will be largely accounted for by compositional differences (in characteristics of carers and care recipients and care arrangements) between sites.

## Method

Survey participants diagnosed with 10/66 dementia and rated as needing care were eligible for this analysis. 10/66 dementia is a cross-culturally calibrated and validated diagnostic algorithm based on cognitive testing, clinical interview and informant report (Prince *et al.*, 2003). Needs for care were identified through open-ended questions to a key informant: Who shares the home? What kind of help does the participant need inside and outside of the home? Who, in the family, is available to care? What help do you provide? Do you help to organize care? Is there anyone else in the family who is more involved in helping? What do they do? What about friends and neighbours, what do they do? The interviewer then coded whether the participant required no care, care some of the time or care much of the time. For the key informant, interviewers were instructed to recruit the person who knew the older person best. Where the older person needed care, then the main carer was selected. Coresidents and family members were prioritized unless others were better qualified. Time spent with the older person was the main criterion if there were several coresident carers. Survey participation was based upon informed consent. Ethical approval was provided by King's College London Research Ethics Committee and by local ethical review boards in each country.

### Characteristics of the person with dementia

Dementia severity was rated with the Clinical Dementia Rating (questionable, mild, moderate or severe) (Morris, 1993) and by cognitive impairment, measured using the Community Screening Instrument for Dementia (CSI‘D’) COGSCORE (Hall *et al.*, 1993). Severity of BPSD was assessed using the brief Neuropsychiatric Inventory (NPI-Q) (Kaufer *et al.*, 2000), administered to an informant. The presence of urinary and/or faecal incontinence was assessed through a single question in the CSI‘D’ informant interview. Mental disorders were identified through a structured clinical mental state interview, the Geriatric Mental State, which applies a computer algorithm [Automated Geriatric Examination for Computer Assisted Taxonomy (AGECAT)] (Copeland *et al.*, 1986) to allocate syndromal diagnoses of organicity (probable dementia), depression, anxiety and psychosis. Stage 1 non-hierarchical diagnoses were used so that all relevant co-morbid conditions could be identified.

### Carer characteristics

Age, sex, marital status (coded for this analysis as currently married versus not currently married), occupation (in paid employment versus economically inactive), relationship to person with dementia (spouse versus child or child-in-law versus other relationship). Carer psychological morbidity was assessed using the Self Reporting Questionnaire [SRQ] (Mari and Williams, 1985).

### Care arrangements

- The extent of care provided (see previous paragraphs) and the time in hours spent by the carer in the last 24 h in specific caregiving activities (Davis *et al.*, 1997); communicating, using transport, dressing, eating, looking after one's appearance and supervising.
- Data on the occupation of the carer, the extent to which the carer had cut back on or stopped work in order to provide care, unpaid care provided by family or others in the community and paid care inputs were assessed using the Client Service Receipt Inventory (Chisholm *et al.*, 2000).

### Carer strain

Carer-perceived strain was assessed using the Zarit Burden Interview (ZBI) (Zarit *et al.*, 1980, 1986; Whitlatch *et al.*, 1991). Twenty-two items assess the carer's appraisal of the impact their involvement has on their lives. It has been very widely used in the USA and Europe and also in Nigeria (Uwakwe and Modebe, 2007), Taiwan (Chou *et al.*, 1999), Korea (Kim *et al.*, 2006), Colombia (Arango Lasprilla *et al.*, 2009) and Argentina (Machnicki *et al.*, 2009). It has been formally validated in China (Ko *et al.*, 2008; Wang *et al.*, 2008) and Japan (Arai *et al.*, 1997). When used in the 10/66 pilot studies in 24 centres in Latin America, India, China and Africa, it was found to be practical, to be culturally relevant and to have robust psychometric properties (10/66 Dementia Research Group, 2004). It was also responsive to change in the context of carer interventions in Russia (Gavrilova *et al.*, 2009), India (Dias *et al.*, 2008) and Peru (Guerra *et al.*, 2011).

### Analyses

We report, by site, numbers and proportion of those with dementia rated as needing care and eligible for inclusion in subsequent analyses. We describe ZBI score distributions in each site (means, standard deviations, medians and interquartile ranges). We assess the univariate effect of each covariate upon ZBI score in each site, reporting stratified ZBI scores with standard deviations and applying *t*-tests or one-way analysis of variance (ANOVA) as appropriate. These were then adjusted for the age and sex of the person with dementia and BPSD severity; the age, sex and marital status of the carer; carer psychological morbidity (SRQ score); the relationship between the carer and care recipient; the time spent providing ADL care; and the number of coresidents. We fitted the model separately for each site and then used a fixed effects meta-analysis to combine site-specific adjusted beta coefficients and their standard errors, reporting adjusted pooled mean differences with 95% confidence intervals and Higgins' *I*^2^ (Higgins and Thompson, 2002) with approximate 95% confidence intervals to estimate the degree of heterogeneity.

## Results

The proportion of those with 10/66 dementia rated as needing care varied widely between sites and, other than in India, was higher in urban than in rural sites. Overall, of the 1345 people with 10/66 dementia, 677 (50.3%) were rated as needing care and were eligible for the care arrangements and carer strain interviews; 673 (99.4%) completed them (Table 1). ZBI scores distributions were only slightly positively skewed in most sites, and the medians were similar to the means. Mean scores for most sites ranged between 17 and 25 with higher mean scores in Cuba (27.9), rural Peru (27.4) and urban China (26.8) (Table 1). The proportion of the variance (eta^2^) in ZBI accounted for by site was 4.1%. The principal characteristics of carers and care recipients in each site are summarized in Tables 2 and 3.

**Table 1:** Sample sizes, response rates, and distribution of Zarit Burden Interview scores by site

Site	Size of base populations and sample			Zarit Burden Interview			
		
	Number of 10/66 dementia cases	Number (%) of dementia cases rated as needing care	Number (%) of eligibles with completed Zarit Burden Interviews	Cronbach's alpha	Median (interquartile range)	Mean (SD)	Estimated mean (95% confidence intervals), after adjustment^a^
Cuba	316	175 (55%)	175 (100%)	0.89	27 (16–39)	27.9 (16.5)	30.5 (27.7–33.3)
Dominican Republic	235	104 (44%)	103 (99%)	0.87	24 (11–36)	24.5 (15.7)	24.3 (20.8–27.8)
Peru (urban)	129	77 (60%)	77 (100%)	0.93	16 (4.5–31)	20.2 (18.3)	22.8 (19.2–26.4)
Peru (rural)	36	12 (33%)	12 (100%)	0.92	29 (12–37)	27.4 (16.4)	24.4 (15.3–33.5)
Venezuela	140	91 (65%)	89 (98%)	0.93	18 (8.5–35.5)	22.7 (17.5)	28.4 (24.0–32.8)
Mexico (urban)	86	48 (56%)	48 (100%)	0.89	17 (9–28)	19.7 (14.4)	23.6 (18.9–28.3)
Mexico (rural)	85	27 (32%)	27 (100%)	0.87	17 (6–30)	18.7 (13.2)	21.2 (15.4–27.1)
China (urban)	81	72 (89%)	72 (100%)	0.95	21 (8–42)	26.8 (20.7)	34.9 (30.6–39.2)
China (rural)	56	28 (50%)	28 (100%)	0.93	16 (2.5–28)	17.1 (14.9)	21.4 (15.5–27.2)
India (urban)	75	15 (20%)	13 (87%)	0.94	20 (8–31)	20.2 (14.9)	20.7 (12.4–29.1)
India (rural)	106	30 (28%)	30 (100%)	0.94	22 (9–30)	21.0 (13.9)	26.2 (20.5–31.9)
Total	1345	677	673			23.8 (17.0)	

a Adjusted for carer age, gender, marital status, relationship to person with dementia and carer psychological morbidity; the age and gender of the person with dementia and severity of behavioural and psychological symptoms of dementia; number of coresidents, time spent assisting with activities of daily living and carer cutting back on work to care (as per model in Table 5).

**Table 2:** The effect of carer characteristics on Zarit Burden Interview scores (*t*-test or ANOVA), with pooled adjusted mean differences by site

Carer characteristics	Cuba, *N* (%)	Dominican Republic, *N* (%)	Peru (urban), *N* (%)	Peru (rural), *N* (%)	Venezuela, *N* (%)	Mexico (urban), *N* (%)	Mexico (rural), *N* (%)	China (urban), *N* (%)	China (rural), *N* (%)	India (urban), *N* (%)	India (rural), *N* (%)	Pooled fixed effect adjusted^a^ mean difference	Test for heterogeneity of estimates
Carer sex													
Female	29.3 (16.6)	24.7 (16.2)	18.2 (16.3)	27.2 (17.2)	23.5 (17.8)	21.1 (14.6)	19.8 (13.6)	26.6 (19.9)	21.1 (15.2)	22.9 (14.5)	20.6 (12.3)	Reference category	
	*N* = 140	*N* = 82	*N* = 66	*N* = 12	*N* = 73	*N* = 42	*N* = 19	*N* = 50	*N* = 15	*N* = 11	*N* = 23		
Male	22.4 (15.5)	23.6 (13.5)	32.5 (25.0)	30.0	17.3 (14.5)	10.5 (9.5)	15.9 (12.6)	27.4 (23.1)	12.5 (13.7)	5.0 (4.2)	22.4 (19.3)	−2.5 (−5.3 to 0.2)	*Q* = 23.1, 10 d.f., *p* = 0.01
	*N* = 35	*N* = 21	*N* = 11	*N* = 1	*N* = 15	*N* = 6	*N* = 8	*N* = 22	*N* = 13	*N* = 2	*N* = 7		*I*^2^ = 57 (15–78)
*T* statistic (*p*-value)	5.0 (0.03)	0.1 (0.79)	6.1 (0.02)	0.0 (0.88)	1.6 (0.21)	2.9 (0.09)	0.5 (0.49)	0.0 (0.88)	2.5 (0.13)	2.8 (0.12)	0.4 (0.76)		
Carer age													
Effect per year of age (SE)	0.18 (0.09)	0.05 (0.10)	0.26 (0.13)	0.20 (0.36)	0.05 (0.14)	−0.02 (0.18)	−0.07 (0.19)	−0.10 (0.16)	−0.18 (0.20)	0.30 (0.28)	0.14 (0.19)	0.00 (−0.08 to 0.07)	*Q* = 38.8, 10 d.f., *p* < 0.001
													*I*^2^ = 74 (53–86)
*T* statistic (*p*-value)	2.1 (0.04)	0.5 (0.60)	1.0 (0.04)	0.5 (0.60)	0.4 (0.71)	−0.1 (0.93)	−0.4 (0.71)	−0.6 (0.53)	−0.9 (0.34)	1.1 (0.31)	0.8 (0.46)		
Carer marital status													
Not currently married	30.6 (17.2)	26.1 (16.7)	19.7 (18.1)	32.8 (19.1)	22.1 (16.5)	22.9 (18.2)	22.0 (16.0)	20.5 (18.4)	1.0	15.7 (18.7)	15.5 (10.6)	Reference category	
	*N* = 60	*N* = 40	*N* = 46	*N* = 5	*N* = 43	*N* = 15	*N* = 6	*N* = 6	*N* = 1	*N* = 3	*N* = 2		
Currently married	27.5 (16.2)	23.8 (15.3)	21.0 (18.7)	23.6 (14.5)	22.8 (18.4)	18.3 (12.5)	17.7 (12.6)	27.4 (21.0)	17.7 (14.8)	21.5 (14.4)	21.4 (14.2)	−3.8 (−6.4 to −1.2)	*Q* = 11.8, 9 d.f., *p* = 0.23
	*N* = 103	*N* = 50	*N* = 31	*N* = 7	*N* = 45	*N* = 33	*N* = 21	*N* = 66	*N* = 27	*N* = 10	*N* = 28		*I*^2^ = 24 (0–63)
*T* statistic (*p*-value)	1.3 (0.26)	0.5 (0.50)	0.1 (0.77)	0.9 (0.36)	0.0 (0.86)	1.1 (0.31)	0.5 (0.50)	0.6 (0.44)	1.2 (0.28)	0.3 (0.57)	0.3 (0.57)		
Carer education													
Effect per level of education (SE)	−0.35 (1.46)	−0.02 (1.38)	−4.03 (2.49)	−6.51 (4.33)	2.11 (2.25)	0.87 (1.70)	3.23 (2.25)	1.13 (1.91)	−1.93 (2.41)	2.22 (3.17)	0.66 (2.39)	1.26 (−0.07 to 2.58)	*Q* = 10.8, 8 d.f., *p* = 0.21
													*I*^2^ = 26 (0–65)
*T* statistic (*p*-value)	0.2 (0.81)	0.0 (0.99)	1.6 (0.11)	1.5 (0.16)	0.9 (0.35)	0.5 (0.61)	1.4 (0.16)	0.6 (0.56)	0.8 (0.43)	0.7 (0.50)	0.3 (0.79)		
Carer occupation													
Paid employment	25.5 (17.7)	23.9 (12.7)	16.9 (18.8)	29.3 (8.1)	21.9 (20.7)	23.4 (19.7)	19.8 (16.5)	23.7(19.8)	16.5 (15.2)	18.3 (9.0)	21.6 (15.1)	Reference category	
	*N* = 66	*N* = 30	*N* = 29	*N* = 3	*N* = 26	*N* = 15	*N* = 10	*N* = 17	*N* = 8	*N* = 3	*N* = 16		
Economically inactive	29.7 (15.5)	24.7 (16.8)	22.2 (17.9)	26.8 (18.8)	22.5 (15.8)	18.1 (11.2)	18.0 (11.4)	27.8 (21.1)	17.3 (15.2)	20.7 (16.6)	20.4 (12.9)	−0.4 (−3.0 to 2.1)	*Q* = 11.2, 9 d.f., *p* = 0.26
	*N* = 108	*N* = 73	*N* = 48	*N* = 9	*N* = 62	*N* = 33	*N* = 17	*N* = 55	*N* = 20	*N* = 10	*N* = 14		*I*^2^ = 20 (0–60)
*T* statistic (*p*-value)	2.6 (0.11)	0.1 (0.82)	1.5 (0.22)	0.1 (0.83)	0.0 (0.88)	1.5 (0.23)	0.1 (0.74)	0.5 (0.47)	0.0 (0.90)	0.1 (0.82)	0.2 (0.82)		
Relation to person with dementia													
Spouse	29.8 (16.4)	29.1 (14.4)	32.4 (18.7)	32.0 (2.8)	25.9 (18.1)	20.3 (8.1)	21.2 (13.4)	25.4 (20.9)	16.4 (14.6)	22.7 (26.0)	26.7 (12.6)	Reference category	
	*N* = 30	*N* = 21	*N* = 10	*N* = 2	*N* = 7	*N* = 4	*N* = 5	*N* = 26	*N* = 12	*N* = 3	*N* = 7		
Child or child-in-law	30.5 (16.4)	25.6 (14.9)	20.4 (17.5)	26.6 (21.9)	23.0 (17.6)	20.2 (15.7)	17.8 (13.3)	28.2 (21.0)	17.6 (15.5)	22.7 (11.9)	20.7 (13.8)	−0.1 (−4.0 to 3.9)	*Q* = 4.1, 9 d.f., *p* = 0.91
	*N* = 98	*N* = 46	*N* = 32	*N* = 7	*N* = 62	*N* = 39	*N* = 16	*N* = 34	*N* = 16	*N* = 6	*N* = 21		*I*^2^ = 0 (0–62)
Other	21.4 (15.3)	20.3 (16.6)	16.6 (17.8)	26.3 (3.8)	20.9 (17.9)	15.6 (5.4)	19.2 (15.1)	26.2 (21.0)		14.5 (11.7)	4.0 (2.8)	−4.9 (−9.7 to −0.05)	*Q* = 4.2, 8 d.f., *p* = 0.84
	*N* = 47	*N* = 36	*N* = 35	*N* = 3	*N* = 18	*N* = 5	*N* = 6	*N* = 12	*N* = 0	*N* = 4	*N* = 2		*I*^2^ = 0 (0–65)
*F* statistic (*p*-value)	5.3 (0.006)	2.4 (0.10)	3.1 (0.05)	0.1 (0.93)	0.2 (0.81)	0.2 (0.80)	0.1 (0.88)	0.1 (0.87)	0.0 (0.85)	0.4 (0.70)	2.3 (0.12)		
Carer psychological morbidity													
Non-case on SRQ (≤7)	25.1 (16.0)	22.0 (15.2)	12.0 (13.1)	36.7 (10.3)	18.5 (16.0)	16.2 (11.9)	16.4 (13.2)	25.7 (19.8)	16.6 (14.9)	23.5 (15.1)	18.8 (10.7)	Reference category	
	*N* = 131	*N* = 72	*N* = 36	*N* = 3	*N* = 71	*N* = 37	*N* = 18	*N* = 70	*N* = 27	*N* = 10	*N* = 25		
Case on SRQ (≥8)	36.4 (15.4)	30.3 (15.5)	27.5 (19.2)	24.3 (17.4)	39.2 (13.0)	31.5 (11.9)	23.2 (12.8)	66.0 (18.4)	29.0	9.0 (7.5)	32.0 (22.9)	9.0 (6.0 to 12.0)	*Q* = 14.0, 8 d.f., *p* = 0.08
	*N* = 44	*N* = 33	*N* = 41	*N* = 9	*N* = 18	*N* = 37	*N* = 9	*N* = 2	*N* = 1	*N* = 3	*N* = 5		*I*^2^ = 43 (0–74)
*T* statistic (*p*-value)	16.7 (<0.001)	6.4 (0.01)	16.7 (<0.001)	1.3 (0.28)	25.7 (<0.001)	11.7 (0.001)	1.6 (0.21)	8.1 (0.006)	0.7 (0.42)	2.5 (0.15)	4.2 (0.05)		
Total *N*													

a Adjusted for carer age, gender, marital status, relationship to person with dementia and carer psychological morbidity; the age and gender of the person with dementia and severity of behavioural and psychological symptoms of dementia; number of coresidents and time spent assisting with activities of daily living.

**Table 3:** The effect of the characteristics of the person with dementia on Zarit Burden Interview scores (*t*-test or ANOVA), with pooled adjusted mean differences by site

Characteristics of the person with dementia	Cuba, *N* (%)	Dominican Republic, *N* (%)	Peru (urban), *N* (%)	Peru (rural), *N* (%)	Venezuela, *N* (%)	Mexico (urban), *N* (%)	Mexico (rural), *N* (%)	China (urban), *N* (%)	China (rural), *N* (%)	India (urban), *N* (%)	India (rural), *N* (%)	Pooled fixed effect adjusted^a^ mean difference	Test for heterogeneity of estimates
Age													
Effect per year of age (SD)	−0.09 (0.16)	−0.43 (0.18)	−0.37 (0.26)	−0.41 (0.80)	−0.02 (0.24)	0.30 (0.30)	−0.22 (0.32)	0.00 (0.39)	−0.37 (0.34)	−0.38 (0.58)	−0.21 (0.34)	0.00 (−0.12 to 0.12)	*Q* = 6.0, 9 d.f., *p* = 0.74
													*I*^2^ = 0 (0–62)
*T* statistic (*p*-value)	−0.57 (0.57)	−2.3 (0.02)	−1.4 (0.16)	−0.5 (0.62)	−0.1 (0.94)	1.0 (0.31)	−0.7 (0.50)	0.0 (1.00)	−1.1 (0.29)	−0.7 (0.52)	−0.6 (0.54)		
Gender													
Female	28.5 (16.5)	21.9 (14.4)	17.9 (16.6)	24.6 (18.2)	21.7 (18.9)	20.6 (15.7)	17.1 (13.9)	27.4 (20.8)	15.2 (14.0)	21.1 (12.7)	18.6 (14.1)	Reference category	
	*N* = 131	*N* = 72	*N* = 53	*N* = 7	*N* = 49	*N* = 35	*N* = 19	*N* = 47	*N* = 14	*N* = 7	*N* = 20		
Male	26.2 (16.9)	30.3 (17.1)	25.5 (20.9)	31.4 (14.5)	25.6 (16.1)	17.2 (9.7)	22.5 (11.4)	25.8 (21.0)	18.9 (16.0)	19.0 (18.3)	25.9 (12.8)	0.3 (−2.6 to 3.2)	10.3 (10 d.f.), *p* = 0.42
	*N* = 44	*N* = 31	*N* = 24	*N* = 5	*N* = 14	*N* = 12	*N* = 8	*N* = 25	*N* = 14	*N* = 6	*N* = 10		*I*^2^ = 3 (0–61)
*T* statistic (*p*-value)	0.6 (0.43)	6.6 (0.01)	2.9 (0.09)	0.5 (0.50)	0.5 (0.49)	0.5 (0.48)	1.0 (0.34)	0.1 (0.75)	0.4 (0.52)	0.1 (0.81)	1.9 (0.18)		
Education													
Effect per level of education (SE)	0.07 (1.24)	−1.65 (1.59)	0.53 (2.19)	0.17 (5.14)	−0.41 (2.83)	1.30 (2.15)	6.40 (2.16)	1.14 (1.82)	−0.39 (2.95)	3.44 (3.25)	4.76 (5.98)	0.60 (−0.72 to 1.93)	6.6 (8 d.f.), *p* = 0.58
													*I*^2^ = 0 (0–65)
*T* statistic (*p*-value)	0.1 (0.96)	1.1 (0.30)	0.2 (0.81)	0.0 (0.97)	0.1 (0.89)	0.6 (0.55)	3.0 (0.007)	0.6 (0.53)	0.1 (0.90)	1.1 (0.31)	0.8 (0.43)		
Depression													
No case level depression on GMS/AGECAT	26.7 (15.6)	21.0 (16.8)	17.6 (17.0)	26.7 (23.2)	19.6 (15.7)	20.3 (14.9)	17.5 (13.2)	25.9 (20.8)	13.6 (13.7)	25.2 (15.1)	24.3 (17.2)	Reference category	
	*N* = 130	*N* = 27	*N* = 56	*N* = 3	*N* = 45	*N* = 22	*N* = 12	*N* = 66	*N* = 21	*N* = 7	*N* = 12		
Case level depression on GMS/AGECAT	31.3 (18.9)	25.7 (15.2)	27.2 (20.1)	27.7 (15.4)	25.9 (18.8)	19.3 (14.3)	19.6 (13.6)	37.2 (18.9)	27.4 (14.4)	14.2 (13.3)	18.8 (11.1)	3.2 (0.4 to 5.9)	*Q* = 8.9, 10 d.f., *p* = 0.55
	*N* = 45	*N* = 76	*N* = 21	*N* = 9	*N* = 44	*N* = 26	*N* = 15	*N* = 6	*N* = 7	*N* = 6	*N* = 18		*I*^2^ = 0 (0–60)
*T* statistic (*p*-value)	2.6 (0.11)	1.8 (0.18)	4.4 (0.04)	0.0 (0.93)	3.0 (0.09)	0.1 (0.81)	0.2 (0.69)	1.6 (0.21)	5.2 (0.03)	1.9 (0.20)	1.2 (0.29)		
Psychosis													
No psychotic symptoms on GMS/AGECAT	26.0 (16.6)	21.8 (14.6)	17.3 (16.7)	25.7 (16.0)	16.5 (14.9)	17.7 (14.2)	18.0 (13.6)	26.1 (21.1)	13.2 (13.5)	21.9 (15.9)	23.0 (13.8)	Reference category	
	*N* = 132	*N* = 51	*N* = 60	*N* = 8	*N* = 55	*N* = 37	*N* = 22	*N* = 67	*N* = 20	*N* = 10	*N* = 26		
Case or subcase level psychosis on GMS/AGECAT	33.7 (15.1)	27.1 (16.4)	30.5 (20.3)	30.8 (19.3)	32.7 (17.0)	26.5 (13.9)	21.4 (12.3)	36.2 (13.9)	26.9 (14.5)	14.3 (11.2)	8.3 (4.3)	8.0 (5.2 to 10.8)	*Q* = 10.4, 10 d.f., *p* = 0.41
	*N* = 43	*N* = 52	*N* = 17	*N* = 4	*N* = 34	*N* = 11	*N* = 5	*N* = 5	*N* = 8	*N* = 3	*N* = 4		*I*^2^ = 4 (0–62)
*T* statistic (*p*-value)	7.1 (0.008)	3.0 (0.09)	7.4 (0.008)	0.2 (0.64)	22.4 (<0.001)	3.3 (0.07)	0.3 (0.62)	1.1 (0.30)	5.7 (0.03)	0.6 (0.46)	4.3 (0.05)		
Anxiety													
No case level anxiety	27.7 (16.4)	24.9 (15.9)	20.0 (18.7)	26.5 (16.9)	20.6 (16.7)	18.2 (13.0)	18.3 (12.6)	26.8 (20.7)	16.1 (14.3)	19.5 (16.2)	20.2 (14.4)	Reference category	
	*N* = 168	*N* = 81	*N* = 68	*N* = 11	*N* = 72	*N* = 45	*N* = 24	*N* = 72	*N* = 27	*N* = 11	*N* = 27		
Case level anxiety on GMS	33.0 (20.5)	23.0 (15.0)	22.2 (15.1)	38.0	31.8 (18.3)	42.3 (19.1)	21.3 (20.8)		42.0	23.5 (0.7)	28.0 (4.4)	7.8 (3.5 to 12.0)	*Q* = 23.8, 9 d.f., *p* = 0.005
	*N* = 7	*N* = 22	*N* = 9	*N* = 1	*N* = 17	*N* = 3	*N* = 3	*N* = 0	*N* = 1	*N* = 2	*N* = 3		*I*^2^ = 62 (25–81)
*T* statistic (*p*-value)	0.7 (0.41)	0.3 (0.61)	0.1 (0.73)	0.4 (0.53)	5.9 (0.02)	9.2 (0.004)	0.1 (0.72)		3.1 (0.09)	0.1 (0.75)	0.8 (0.37)		
CDR													
Effect per level of CDR (SD)	3.6 (1.4)	5.0 (1.7)	6.4 (2.7)	3.6 (5.8)	4.9 (2.8)	5.9 (3.3)	−0.3 (4.3)	6.1 (3.9)	9.5 (3.6)	10.5 (4.5)	10.9 (2.9)	2.8 (1.1 to 4.5)	*Q* = 5.4, 10 d.f., *p* = 0.86
													*I*^2^ = 0 (0–60)
*T* statistic (*p*-value)	2.5 (0.01)	2.9 (0.01)	2.4 (0.02)	0.6 (0.56)	1.8 (0.08)	1.8 (0.08)	−0.1 (0.95)	1.6 (0.12)	2.6 (0.02)	2.3 (0.04)	3.8 (0.001)		
Severity of behavioural and psychological symptoms of dementia													
Effect per point on the NPI-Q total severity score (SD)	0.81 (0.23)	1.14 (0.17)	1.27 (0.31)	1.50 (0.81)	1.91 (0.23)	0.98 (0.32)	0.44 (0.38)	0.64 (0.63)	1.35 (0.60)	1.65 (0.60)	1.27 (0.37)	0.98 (0.79 to 1.17)	*Q* = 21.9, 9 d.f. *p* = 0.009
													*I*^2^ = 59 (17–80)
*T* statistic (*p*-value)	3.6 (<0.001)	6.6 (<0.001)	4.1 (<0.001)	1.8 (0.10)	8.3 (<0.001)	3.1 (0.004)	1.2 (0.26)	1.0 (0.31)	2.3 (0.03)	2.8 (0.02)	3.4 (0.002)		
Cognitive function													
Effect per point on the CSI‘D’ total COGSCORE (SD)	−0.26 (0.14)	−0.25 (0.19)	0.11 (0.19)	0.61 (0.60)	−0.46 (0.21)	−0.08 (0.28)	0.10 (0.33)	−0.21 (0.23)	−0.34 (0.30)	−0.69 (0.34)	−0.65 (0.24)	−0.02 (−0.16 to 0.11)	*Q* = 4.3, 8 d.f. *p* = 0.83
													*I*^2^ = 0 (0–65)
*T* statistic (*p*-value)	−1.8 (0.07)	−1.3 (0.19)	0.6 (0.56)	1.0 (0.34)	−2.2 (0.03)	−0.3 (0.77)	0.3 (0.76)	−0.9 (0.37)	−1.2 (0.26)	−2.0 (0.07)	−2.7 (0.01)		
Incontinence													
No incontinence	26.9 (17.0)	22.4 (14.4)	16.2 (17.6)	27.2 (18.2)	19.5 (16.6)	18.1 (14.5)	17.2 (13.3)	17.7 (16.7)	5.9 (11.8)	15.3 (13.9)	17.1 (10.9)	Reference category	
	*N* = 89	*N* = 52	*N* = 36	*N* = 10	*N* = 54	*N* = 35	*N* = 17	*N* = 33	*N* = 12	*N* = 4	*N* = 22		
Incontinence	29.5 (16.0)	28.2 (18.6)	26.6 (22.5)	28.0	30.5 (20.0)	28.0 (18.0)	15.5 (3.5)	36.4 (19.6)	22.8 (9.0)	27.8 (16.6)	53.0 (15.6)	2.4 (−1.1 to 5.8)	*Q* = 6.8, 8 d.f., *p* = 0.56
	*N* = 74	*N* = 29	*N* = 17	*N* = 1	*N* = 14	*N* = 3	*N* = 2	*N* = 16	*N* = 6	*N* = 5	*N* = 2		*I*^2^ = 0 (0–65)
*T* statistic (*p*-value)	1.0 (0.32)	1.5 (0.13)	1.8 (0.07)	-	2.1 (0.04)	1.1 (0.27)	0.2 (0.86)	3.5 (0.001)	3.1 (0.007)	1.2 (0.26)	4.4 (<0.001)		

GMS, Geriatric Mental State; AGECAT, Automated Geriatric Examination for Computer Assisted Taxonomy; CDR, clinical dementia severity.

a Adjusted for carer age, gender, marital status, relationship to person with dementia and carer psychological morbidity; the age and gender of the person with dementia and severity of behavioural and psychological symptoms of dementia; number of coresidents and time spent assisting with activities of daily living.

### Carer characteristics

Neither carer age nor occupation was associated with strain. Married carers reported less strain than non-married carers; although this effect was not statistically significant in any site, the pooled mean difference was substantial (−3.7, 95% confidence intervals −6.4 to −1.2) without significant heterogeneity across sites. There was also a consistent effect across sites for carers who were neither children nor spouses to report lower levels of strain compared with spousal carers. Children and children-in-law reported similar levels of strain to spousal carers. Carer psychological morbidity was strongly associated with carer strain in most sites. The meta-analysed mean difference was large (9.0, 95% confidence intervals 6.0 to 12.0), but there was minor heterogeneity with an association in the reverse direction in rural India.

### Characteristics of the person with dementia

Neither age nor sex was associated with carer strain. Co-morbid depression, psychosis and anxiety were each associated with carer strain. The adjusted meta-analysed mean differences were larger for psychosis (8.0, 95% CI 5.2 to 10.8) and anxiety (7.8, 95% CI 3.5 to 12.0) than for depression (3.2, 95% CI 0.4 to 5.9). None of these effects was adjusted for BPSD, as the NPI-Q scale includes items that assess the presence and severity of depression, hallucinations and delusions. There was moderate heterogeneity across sites for the effect of anxiety; however, this was in the size of the positive effect rather than the direction of association. Clinical severity of dementia (CDR) was also linearly positively associated with carer strain, with no heterogeneity across sites. BPSD severity (NPI-Q severity scale) was also linearly positively associated with carer strain, with approximately a one-point increase in ZBI score for every one point increase in NPI-Q severity (0.98, 95% CI 0.79 to 1.17). There was moderate heterogeneity but, again, only in the size of the positive effect. Only in three sites, Cuba, Venezuela and rural India, was there a significant univariate association between more impaired cognitive function (COGSCORE) and carer strain. After adjusting for covariates, including BPSD severity, the effect was consistently null across sites (−0.02, 95% CI −0.16 to 0.11).

### Care arrangements

The pooled mean difference contrasting those needing ‘much care’ with those needing some care was 4.3, 95% CI 1.6 to 6.9, with no significant heterogeneity across sites (Table 4). Time spent assisting with ADL care was also associated with carer strain with a one-point increase in ZBI score for every one additional hour spent on ADL care (1.1, 95% CI 0.75 to 1.45) but with some heterogeneity in the size of the association across sites. Mean ZBI scores were higher for carers who had cut back or stopped work to care in all sites except for urban India, to a statistically significant degree in six of 11 sites. The pooled adjusted mean difference was 6.7, 95% CI 4.0 to 9.4. Paid carers were only common in Cuba, the Dominican Republic, urban Peru, Venezuela and urban China. In the first three of those sites, mean ZBI scores were lower among those who had used paid carers, whereas in Venezuela and urban China, carer strain was a little higher in that group. The adjusted pooled mean difference tended in the direction of less strain among those using paid carers (−3.2, 95% CI −6.7 to 0.2). A similar trend was observed for those who had made use of additional informal care (−2.2, 95% CI −4.7 to 0.4). More coresidents was not associated with a reduction in carer strain, although there was a trend in this direction with a half-point reduction in ZBI score for each additional resident (−0.52, 95% CI −1.16 to 0.11) and a large statistically significant linear effect in urban China.

### Final model

The final model incorporating pooled data from all sites accounted for 33.7% of variance in carer strain, with 7.5% accounted for by site, 5.7% by carer characteristics (principally psychological morbidity), 12.0% by characteristics of the person with dementia (principally BPSD severity) and 8.7% by care arrangements (principally time assisting with ADL care and carer giving up work to care). Thus, the variance accounted for by site increased after adjusting for compositional differences in the characteristics of the carer, the person with dementia and the nature of the care arrangements. After adjusting for these factors, carer strain was highest in urban China, in Cuba and in Venezuela and lowest in rural Mexico, in rural China and in urban India (Table 1).

**Table 4:** The effect of care arrangements on Zarit Burden Interview scores (*t*-test or ANOVA), with pooled adjusted mean differences by site

Care arrangements	Cuba, *N* (%)	Dominican Republic, *N* (%)	Peru (urban), *N* (%)	Peru (rural), *N* (%)	Venezuela, *N* (%)	Mexico (urban), *N* (%)	Mexico (rural), *N* (%)	China (urban), *N* (%)	China (rural), *N* (%)	India (urban), *N* (%)	India (rural), *N* (%)	Pooled fixed effect adjusted^a^ mean difference	Test for heterogeneity of estimates
Needs for care (rated by interviewer)													
‘Some care’	25.0 (17.6)	17.3 (16.5)	16.0 (18.3)	24.3 (16.3)	15.4 (14.1)	18.1 (13.5)	19.8 (14.0)	17.8 (16.8)	2.8 (3.4)	6.0 (5.3)	15.8 (11.6)	Reference category	
	*N* = 43	*N* = 23	*N* = 28	*N* = 6	*N* = 39	*N* = 17	*N* = 13	*N* = 15	*N* = 10	*N* = 3	*N* = 20		
‘Much care’	28.9 (16.1)	26.5 (14.9)	22.7 (18.0)	30.5 (17.5)	28.4 (17.9)	20.7 (15.1)	17.6 (12.9)	29.2 (21.1)	25.0 (12.6)	24.4 (14.2)	31.4 (12.5)	4.3 (1.6 to 6.9)	*Q* = 9.1, 8 d.f., *p* = 0.34
	*N* = 132	*N* = 80	*N* = 77	*N* = 6	*N* = 50	*N* = 31	*N* = 14	*N* = 57	*N* = 18	*N* = 10	*N* = 10		*I*^2^ = 12 (0–54)
*T* statistic (*p*-value)	1.8 (0.18)	6.4 (0.01)	2.4 (0.13)	0.4 (0.54)	13.9 (<0.001)	0.4 (0.56)	0.2 (0.66)	3.7 (0.06)	29.2 (<0.001)	4.6 (0.06)	11.4 (0.002)		
Time spent assisting with ADL													
Effect per hour of ADL assistance (SE)	0.84 (0.31)	0.58 (0.49)	2.56 (0.7)	0.65 (1.85)	0.97 (0.53)	0.80 (0.70)	0.13 (0.81)	1.58 (0.56)	3.04 (0.46)	1.36 (0.87)	3.69 (1.81)	1.10 (0.75 to 1.45)	*Q* = 30.4, 9 d.f., *p* < 0.001
													*I*^2^ = 70 (43–85)
*T* statistic (*p*-value)	2.8 (0.006)	1.3 (0.24)	3.7 (<0.001)	0.4 (0.73)	1.8 (0.07)	1.2 (0.26)	0.2 (0.87)	2.8 (0.006)	6.7 (<0.001)	1.6 (0.15)	2.0 (0.05)		
Household assets													
Effect per asset (SE)	0.77 (1.16)	−1.35 (1.11)	0.51 (4.89)	−6.65 (2.52)	−8.25 (3.94)	−0.32 (1.74)	−0.03 (1.31)	5.26 (3.78)	−0.09 (1.93)	3.04 (2.02)	−0.96 (1.89)	0.29 (−0.86 to 1.43)	*Q* = 5.6, 8 d.f., *p* = 0.70
													*I*^2^ = 0 (0–65)
*T* statistic (*p*-value)	0.6 (0.51)	1.2 (0.23)	0.1 (0.92)	2.6 (0.03)	2.1 (0.04)	0.2 (0.86)	0.2 (0.99)	1.4 (0.17)	0.1 (0.96)	1.5 (0.16)	0.5 (0.62)		
Carer cutback work to care													
Carer has not cut back on work	27.5 (16.6)	21.7 (15.4)	17.2 (16.2)	25.5 (14.0)	17.9 (14.9)	18.0 (13.8)	17.1 (14.4)	25.5 (20.6)	9.3 (11.6)	22.0 (11.9)	15.7 (11.4)	Reference category	
	*N* = 114	*N* = 71	*N* = 69	*N* = 8	*N* = 69	*N* = 37	*N* = 17	*N* = 67	*N* = 11	*N* = 4	*N* = 19		
Carer has cut back or stopped work to care	28.6 (16.6)	30.6 (14.7)	46.1 (15.0)	31.3 (22.5)	39.4 (15.8)	25.7 (15.7)	21.4 (11.2)	44.2 (15.2)	22.1 (14.9)	19.3 (16.6)	30.1 (13.5)	6.7 (4.0 to 9.4)	*Q* = 29.5, 8 d.f., *p* < 0.001
	*N* = 61	*N* = 32	*N* = 8	*N* = 4	*N* = 20	*N* = 11	*N* = 10	*N* = 5	*N* = 17	*N* = 9	*N* = 11		*I*^2^ = 73 (47–86)
*T* statistic (*p*-value)	0.2 (0.69)	7.6 (0.007)	23.1 (<0.001)	0.3 (0.59)	31.4 (<0.001)	2.5 (0.12)	0.7 (0.42)	3.9 (0.05)	5.9 (0.02)	0.1 (0.78)	9.7 (0.004)		
Paid care													
No paid care	28.7 (16.6)	25.9 (12.7)	23.5 (15.8)	27.3 (17.2)	22.4 (17.4)	19.1 (14.3)	18.9 (13.4)	23.3 (17.6)	17.1 (14.9)	20.2 (14.9)	21.0 (13.9)	Reference category	
	*N* = 149	*N* = 80	*N* = 48	*N* = 11	*N* = 77	*N* = 46	*N* = 26	*N* = 34	*N* = 28	*N* = 13	*N* = 30		
Paid care	23.5 (15.9)	19.3 (12.7)	14.8 (15.8)	29.0	25.0 (18.9)	35.4 (3.3)	13.0	30.0 (22.9)				−3.2 (−6.7 to 0.2)	*Q* = 6.9, 6 d.f., *p* = 0.33
	*N* = 26	*N* = 23	*N* = 29	*N* = 1	*N* = 12	*N* = 2	*N* = 1	*N* = 38	*N* = 0	*N* = 0	*N* = 0		*I*^2^ = 13 (0–75)
*T* statistic (*p*-value)	2.2 (0.14)	3.2 (0.08)	4.3 (0.04)	0.0 (0.93)	0.2 (0.63)	2.5 (0.12)	0.2 (0.67)	1.9 (0.17)	-	-	-		
Unpaid (informal) care													
No additional informal care	30.4 (18.2)	23.3 (16.0)	23.9 (20.3)	24.2 (20.5)	21.4 (16.6)	24.9 (15.3)	18.9 (15.6)	26.9 (21.1)	16.6 (15.0)	17.6 (11.2)	21.1 (12.2)	Reference category	
	*N* = 86	*N* = 55	*N* = 42	*N* = 5	*N* = 37	*N* = 19	*N* = 9	*N* = 66	*N* = 20	*N* = 8	*N* = 9		
Additional informal care	26.0 (14.3)	25.5 (15.3)	15.8 (14.6)	29.7 (14.2)	23.7 (18.2)	16.3 (13.0)	18.6 (12.4)	26.7 (17.2)	18.4 (15.6)	24.2 (20.2)	21.0 (14.8)	−2.2 (−4.7 to 0.4)	*Q* = 10.4, 8 d.f., *p* = 0.24
	*N* = 86	*N* = 47	*N* = 35	*N* = 7	*N* = 52	*N* = 29	*N* = 18	*N* = 6	*N* = 8	*N* = 5	*N* = 21		*I*^2^ = 23 (0–64)
*T* statistic (*p*-value)	3.1 (0.08)	0.5 (0.48)	3.9 (0.05)	0.3 (0.59)	0.4 (0.55)	4.4 (0.04)	0.0 (0.95)	0.0 (0.98)	0.1 (0.78)	0.6 (0.46)	0.0 (0.98)		
Number of coresidents													
Effect per additional coresident (SE)	−0.93 (0.78)	0.22 (0.77)	−0.58 (1.05)	−1.06 (1.63)	−1.02 (0.75)	0.16 (0.92)	−0.08 (1.42)	−3.60 (1.57)	0.67 (1.59)	2.45 (2.75)	−0.95 (1.14)	−0.52 (−1.16 to 0.11)	*Q* = 6.4, 8 d.f., *p* = 0.60
													*I*^2^ = 0 (0–65)
*T* statistic (*p*-value)	−1.2 (0.23)	0.3 (0.78)	−0.6 (0.58)	−0.7 (0.53)	−1.4 (0.18)	0.2 (0.87)	−0.1 (0.96)	−2.3 (0.02)	0.4 (0.68)	0.9 (0.39)	−0.8 0.41		

a Adjusted for carer age, gender, marital status, relationship to person with dementia and carer psychological morbidity; the age and gender of the person with dementia and severity of behavioural and psychological symptoms of dementia; number of coresidents and time spent assisting with activities of daily living.

### Discussion

A recent meta-analysis of studies of correlates of carer strain identified 228 studies, 89% using convenience samples (Pinquart and Sorensen, 2003). Associations with carer strain were more marked in representative samples, perhaps because of over-representation of severe dementia and stressed carers in convenience samples. The average sample size for most exposure/outcome combinations did not exceed 150 and was often much less than this. With 673 carer/care recipient dyads from 11 sites in seven countries, ours is therefore one of the largest studies of strain among dementia carers and adds significantly to the limited evidence from LMIC. Furthermore, we have recruited representative population-based samples of people with dementia, with high response rates. We acknowledge several important limitations. Whereas the overall sample size was large, numbers in individual sites were sometimes small, and there may have been important heterogeneity of effects in the meta-analysis not captured by the relatively underpowered statistical tests. Although we studied many correlates of carer strain, several potentially important mediators were not assessed, including family relationships, premorbid quality of the relationship between carer and care recipient, carer personality and carer coping strategies. This may have explained the relatively low proportion of the variance in carer strain accounted for in the final model.

Mean ZBI scores ranged between 17.1 and 27.9 by site, lower than those for our pilot study convenience samples, which were between 23 and 37 for the majority of sites (10/66 Dementia Research Group, 2004). The mean ZBI score for the EUROCARE study convenience samples was 36, with country means ranging between 28 and 52. In the systematic review of correlates of carer strain, the mean ZBI score was 29.9; again, the large majority of these studies used convenience samples, among which carer strain is likely to be higher.

Consistent with previous research (Yee and Schulz, 2000), female carers reported higher levels of strain, independent of the relationship to the person with dementia, the characteristics of the persons with dementia and care arrangements. Our hypothesis that spouse carers would report more strain was not supported. There is scant evidence to support this association from HIC studies (Sorensen *et al.*, 2006), and cultural specificity is quite likely. The role of daughters-in-law has been extensively studied in Korea, with inconsistent findings regarding their experience of strain compared with that of children and spouses (Lee and Sung, 1997; Hong and Kim, 2008). In our study, only in China and in India were children-in-law sufficiently often identified as main carers to be studied as a separate group, and no significant differences were seen when compared with spouses and children.

The most substantial correlates of carer strain were the primary stressors BPSD, dementia severity, needs for care and time spent caring. The assessment of the extent of needs for care was pragmatic, with good face validity, but we did not have the opportunity to pre-assess validity or reliability across raters and countries. The additional strain associated with needing ‘much care’ supports its concurrent validity. Consistent with our hypothesis, the effects of BPSD were more prominent than those of cognitive impairment. Similar findings have been reported from studies in China (Wang *et al.*, 2008), Argentina (Machnicki *et al.*, 2009) and Colombia (Arango Lasprilla *et al.*, 2009). When psychological symptoms were considered separately, there were independent effects of psychosis, anxiety and depression, assessed through structured clinical interview of the person with dementia, using the Geriatric Mental State. As in our pilot study (Ferri *et al.*, 2004), effects of anxiety and psychosis were more prominent than those of depression. We also explored the effects of incontinence, given suggestions from qualitative research in India of its burdensome and stigmatizing nature (Shaji *et al.*, 2002), and the high prevalence among people with dementia in our sample (Prince *et al.*, 2011). Carer strain was consistently higher where the care recipient was incontinent. However, this effect was substantially confounded by BPSD in the pooled multivariable analysis.

There was no consistent evidence for an association between socioeconomic status and carer strain. In the univariate analysis, household assets were inversely associated with strain in rural Peru and Venezuela, with a strong but non-significant trend in the opposite direction in urban sites in China and India. Neither was there any consistent evidence to support associations between level of education, for the carer or the person with dementia, and carer strain. However, cutting back on work to care was significantly associated with higher carer strain in most sites with trends in the same direction in others. The meta-analysed effect size of 6.7 points on the ZBI was substantial.

Paid carers were relatively common only in the urban Latin American sites and in urban China, with no paid care reported in rural China or in India. In Latin America, the use of paid carers was generally associated with lower levels of strain for the main family carer. However, in urban China, there was a non-significant trend in the opposite direction. The introduction of the Long Term Care insurance scheme in Japan has shown that even in bastions of traditional family care, paid homecare can alleviate carer strain (Kumamoto *et al.*, 2006). However, paid care in Latin America and in China is overwhelmingly informal and unregulated, with untrained and inexperienced care workers, usually recruited from among rural migrants to cities. We also found a non-significant overall trend towards lower levels of strain among carers who reported additional informal support from other family members or friends. These effects were more prominent in Latin American sites, and indeed, few Chinese or Indian carers reported receiving additional informal support.

Variation in mean carer strain was not accounted for by compositional differences among sites. In fact, after adjusting for a range of relevant factors—carer age, sex, marital status, relationship to person with dementia and carer psychological morbidity; the age and sex of the person with dementia and BPSD severity; number of coresidents, time spent providing care and carer cutting back on work to care—the variance component accounted for by site increased from 4.1% to 7.5%, with particularly high adjusted marginal mean scores in Cuba and urban China. The reasons for these outliers are not immediately obvious, although urban China stood out in having a particularly high proportion of paid carers (associated in that site with higher family carer strain) and a low proportion of carers giving up work to care. Holding dual positions of worker and carer could lead to role strain or alternatively provide an outlet to help carers better manage the demands placed upon them (Edwards *et al.*, 2002).

Our findings underline the impact of caring for a person with dementia in LMIC and the need for scaling up carer support, education and training interventions (Prince *et al.*, 2009). We have recently reported benefits on outcomes including carer strain and psychological morbidity in randomized controlled trials of the 10/66 Dementia Research Group's ‘Helping Carers to care’ intervention in India, Russia and Peru (Dias *et al.*, 2008; Gavrilova *et al.*, 2009; Guerra *et al.*, 2011). Our finding that giving up work to care was both prevalent and associated with substantial increased strain emphasizes the adverse economic impact of taking up a caring role on the main carer and the whole household. The lack of carer compensatory benefits and limited access to disability benefits should be a concern for policymakers (Prince *et al.*, 2008).
